# Student Counseling Centers in Europe: A Retrospective Analysis

**DOI:** 10.3389/fpsyg.2022.894423

**Published:** 2022-04-22

**Authors:** Isabella Giulia Franzoi, Maria Domenica Sauta, Giuliano Carnevale, Antonella Granieri

**Affiliations:** Department of Psychology, University of Turin, Turin, Italy

**Keywords:** emerging adulthood, Europe, mental health, students, student counseling centers

## Abstract

**Objective:**

Tertiary education can be stressful for many young people, who consistently report high levels of distress. The issue has major implications for campus health services and mental health policymaking more widely. The present study proposes to map student counseling services in Europe.

**Methods:**

The sample of institutions was sourced, using standardized data extraction, from the European Tertiary Education Register (ETER). Then, each institution’s website was analyzed for information about the availability of student counseling centers and the services provided. Data extracted from the ETER database were: ETER ID, national identifier, institution name, English institution name, number of students, legal status (in English), institution category (in English), and institutional website. Data extracted from institutions’ websites concerned the availability of students’ psychological centers and the services provided. Analyses were carried out using the SPSS Statistics software package (IBM Corp., Armonk, NY, United States), version 26.

**Results:**

Overall, it was found that most institutions do not provide mental health counseling services for their students. Institutions of medium dimensions showed a higher probability of reporting students’ psychological centers than small institutions. Moreover, private institutions and public institutions were more likely to report having such centers, while private government-dependent institutions were less likely. Universities of applied sciences and universities were more likely to report having them, while other institutions were less likely. Regarding provision according to geographic area, compared to Northern Europe, every other European region was less likely to report featuring such centers. Most institutions reported offering counseling, career counseling, or not otherwise specified psychological services, but only a small number reported providing services such as psychotherapy, psychiatric services, or counseling for learning-specific disabilities.

**Conclusion:**

It is critically important to catalog European data on student counseling centers and services, to encourage tertiary education institutions to invest in such services as key sites for mental health promotion. Indeed, professionally trained staff and the possibility of long-term treatment options would go a long way in supporting students who might not otherwise have access to treatment.

## Introduction

For many young people in their 20s, the transitional period between adolescence and adulthood is the age of tertiary education. Attending university or another higher education institution can be stressful for many of them, as it often implies several significant changes in their living conditions and lifestyles, and facing challenging tasks such as career choice, relocation, and academic demands (Credé and Niehorster, 2012; Schulenberg and Schoon, 2012; Arnett, 2016). Several prior studies have concluded that the tertiary education years represent a crucial period for the mental health of emerging adults (Ozer et al., 2019; Lei et al., 2021), who have been found to consistently report higher levels of distress than those reported by the general population (James et al., 2017; Tariku et al., 2017; Cvetkovski et al., 2019; Franzoi et al., 2021; Granieri et al., 2021). Such findings are of great concern for educational systems worldwide (Cvetkovski et al., 2018; Lo et al., 2020; Kalkbrenner et al., 2020). Correspondingly, psychological distress can interfere significantly with students’ personal and academic lives, and particularly affects young people from lower socioeconomic backgrounds (Collins and Mowbray, 2005; Eisenberg et al., 2007; Fradelos et al., 2019).

The tertiary education years are characterized by many potential stressors, such as housing conditions and financial problems (Flett et al., 2009; Tosevski et al., 2010; Yasar and Turgut, 2020), and, for many students, this is their first experience away from their family, which may make adapting to a new social context even more difficult (Monti et al., 2013). Moreover, students often have to face increased study demands, and, therein, a greater pressure to succeed, feelings of inadequacy, competition with peers, and insecurity about the future (Macaskill, 2013; Kreß et al., 2015). Several studies point to high levels of depression, anxiety, and suicide risk in tertiary education students (Beiter et al., 2015; Rotenstein et al., 2016; Oyekcin et al., 2017; Poorolajal et al., 2017; Tang et al., 2018; Franzoi et al., 2020). Moreover, they can experience personal and relational problems that may indirectly affect their academic careers, often slowing down or even occasionally ending them, or preventing these students’ social integration or academic engagement. Certainly, it is well known that students suffering higher levels of psychological distress also experience an increased risk of academic failure and dropping out (Jaisoorya et al., 2017; Ishii et al., 2018).

These data on students mental health and academic performances have major implications for campus health services and mental health policymaking more widely (Viñas Poch et al., 2004; Farrer et al., 2019), with student counseling centers found to provide a unique source of guidance and support for these young people (Gallagher, 2009; Buchanan et al., 2012). Counseling services have been established in several tertiary education institutions around the world (Kraft, 2009); in Europe, student counseling services are characterized by different approaches, but the most common clinical models comprise psychodynamic, cognitive–behavioral, and humanistic approaches (Rückert, 2015). Monti et al. (2014) observed that both psychodynamic and cognitive–behavioral interventions were effective in reducing psychological distress among university students. In addition, since such counseling centers are located in a protected and familiar context for students, they feel better able to overcome the contextual, intrapersonal, and interpersonal issues that often prevent them from asking for psychological support.

The central role of student counseling centers is accentuated by previous data showing a progressive increase in the severity of the issues treated in such contexts (Storrie et al., 2010; Holm-Hadulla and Koutsoukou-Argyraki, 2015; Biasi et al., 2017). Furthermore, research has shown that these interventions maintain their effectiveness over time, with benefits for the students’ long-term mental health as well as their academic career (Monti et al., 2016; Cerutti et al., 2020). Conversely, untreated psychological problems have been found to have a negative impact on academic performance, retention, and graduation rates, leading not only to academic consequences, such as dropping out of university, but also to more severe mental health problems such as suicidal behavior, drug addiction, and alcoholism (Monk, 2004; Koutra et al., 2010; Pillay, 2021).

Student counseling services have the dual task of both supporting young adults experiencing difficulties and also giving feedback to the academic establishment (Adamo et al., 2012). Moreover, while such counseling interventions are often aimed at tackling the ongoing issues that are affecting a distressed student’s academic performance, they can also represent a first step for students with a stable psychopathology, allowing them access to mental health services or psychotherapeutic interventions (Biasi et al., 2017).

For these reasons, we think that a fist comprehensive overview of data available online on student counseling services in Europe is of fundamental importance, as a first step to gain a better comprehension of the interventions provided, how these services are accessed, and the major issues affecting students’ mental health. In the United States, since 1920, the American College Health Association has collected this type of information regarding students’ health and wellbeing. Over 800 higher education institutions are members of the Association, representing the collective health and wellbeing needs of about 10 million college students.

However, as far as we know, there has to date been no similar research completed regarding the availability of student counseling services in European tertiary education institutions. We wondered if the differences regarding the provision of psychological services in European Tertiary Education are linked to socio-economic, legal or social and health organization issues.

Recent years have been characterized by a decreasing of funding for public services. In the higher education sector, institutions have to prove higher education to a growing population without an increasing in government funding. In particular, in the last half century, the private sector of higher education has grown very rapidly (Levy, 2012). Aspects distinguish the private sector from the public (i.e., the financial contribution, which must derive especially from private sources, contrary to the public sector and a stronger autonomy in relation to decisions on financial policy) may have a role in providing psychological services. Moreover, may be differences regarding the size of universities: it is possible that smaller universities have less funds than larger ones. In the view of this considerations it would be interesting assessing whether these differences occur within each single European area. Thus, the present study is a retrospective analysis of data available online pertaining to student counseling centers in Europe. In particular, our concern is exploring (a) the presence or absence of students’ psychological services in different European areas; (b) the presence or absence of students’ psychological services in different geographical areas, according to an institution’s dimension, legal status and category; (c) if institution’s dimension, legal status, category, and the geographical area have effects on the likelihood that will have a student psychological center; and (d) the types of interventions that are provided in these centers.

## Materials and Methods

### Data Collection

To assess the presence of students’ psychological centers and the services provided by them across Europe, we used a two-step search strategy. First, we searched the European Tertiary Education Register (ETER) (eter-project.com) to identify tertiary education institutions throughout the European area, including the 28 member states of the European Union (EU) at the time of our search, candidate countries, and potential candidates, as well as other countries in the European geographical area as long as they were featured in the ETER. The search interrogated 2016 and 2017 data, the latest that were available at the time the search was completed (the last search for any updates was carried out on September 1, 2020). A unique database was then created that combined 2016 and 2017 data and eliminated any duplicates.

Next, we searched each of our sample institutions’ websites to collect information about the availability of student counseling centers and the services provided. When available, we screened the English version of the websites; otherwise, we screened the national language’s version. The website searches were performed by Authors [IF, MS, and GC]; each website available was independently accessed by two of them, and in cases of disagreement regarding data extraction from the websites the third author was consulted.

Data analysis was carried out by utilizing a standardized data extraction form. The following data were extracted from the ETER database: ETER ID, national identifier, institution name, English institution name, number of students, legal status (in English), institution category (in English), and institutional website. Data extracted from institutions’ websites concerned the availability of students’ psychological centers and the services provided. In particular, we coded through a dichotomic variable if each institution provided: unspecified psychological services, counseling services, psychotherapy services, psychiatric services, psychological services, career counseling, group counseling, counseling for disabled students, psychoeducational counseling, counseling for foreign students, sport psychology counseling, nutritional counseling, spiritual counseling, social counseling, health counseling, sexual counseling, counseling for learning specific difficulties, phone counseling and language counseling. Then, we added a category to cover the subregions of the European area based on the classifications provided by the United Nations geoscheme for Europe: Eastern Europe, Northern Europe, Southern Europe, and Western Europe. Turkey and Cyprus, which are included in the ETER, but are grouped into Western Asia in the United Nations geoscheme, were coded in this study as Middle Eastern Europe. Moreover, we added a category to discriminate between small institutions (up to 10,000 students), medium institutions (from 10,000 to 20,000 students), large institutions (from 20,000 to 40,000 students), and very large institutions (more than 40,000 students), following the categories provided by the Italian Centre for Social Investment Studies.

The process of extracting data from the ETER database resulted in 2,985 institutions. Table 1 shows the number of institutions mapped for every country and the countries included in every geographical area. Data will be presented as being grouped by geographical area.

**TABLE 1:** Descriptive statistics of the institutions mapped in each country for every geographical area.

*Geographical area*		*N*	*%*
Northern Europe	Denmark	33	5.97
	Estonia	25	4.52
	Finland	41	7.41
	Iceland	7	1.27
	Ireland	25	4.52
	Latvia	44	7.96
	Lithuania	43	7.78
	Norway	38	6.87
	Sweden	37	6.69
	UK	260	47.02
	*Total*	*553*	*100.00*
Eastern Europe	Bulgaria	52	9.17
	Czech Republic	65	11.46
	Hungary	53	9.35
	Poland	270	47.62
	Romania	95	16.75
	Slovakia	32	5.64
	*Total*	*567*	*100.00*
Western Europe	Austria	70	6.90
	Belgium	63	6.21
	France	389	38.33
	Germany	400	39.41
	Liechtenstein	1	0.10
	Luxembourg	2	0.20
	Netherlands	55	5.42
	Switzerland	35	3.45
	*Total*	*1,015*	*100.00*
Southern Europe	Albania	40	6.22
	Croatia	37	5.75
	Greece	47	7.31
	Italy	216	33.59
	Macedonia	16	2.49
	Malta	2	0.31
	Montenegro	10	1.56
	Portugal	95	14.77
	Serbia	46	7.15
	Slovenia	52	8.09
	Spain	82	12.75
	*Total*	*643*	*100.00*
Middle Eastern Europe	Cyprus	26	12.56
	Turkey	181	87.44
	*Total*	*207*	*100.00*
**Total**		**2,985**	**100**

Our database search obtained 553 tertiary education institutions in Northern Europe, 567 in Eastern Europe, 1,015 in Western Europe, 643 in Southern Europe, and 207 in Middle Eastern Europe, for a total of 2,985 institutions mapped.

### Data Analysis

Data were coded and analyzed using the SPSS Statistics software package (IBM Corp., Armonk, NY, United States), version 26. The statistical significance was set at *p* ≤ 0.05. We performed descriptive statistics on data collected for each country and each European area. Differences in the presence versus absence of students’ psychological centers according to an institution’s dimension, legal status and category in different geographical areas were assessed through Chi-square tests. A logistic regression was performed to ascertain the effects of an institution’s dimension, legal status, category and geographical area on the likelihood that the institution would have a psychological center.

## Results

### Institution Dimension

As detailed in Table 2, in Northern Europe, we mapped 368 (66.55%) small institutions, 103 (18.63%) medium institutions, 52 (9.40%) large institutions, and 2 (0.36%) very large institutions. In Eastern Europe, we found listings for 398 (70.19%) small institutions, 42 (7.41%) medium institutions, 24 (4.23%) large institutions, and 2 (0.35%) very large institutions. Regarding Western Europe, there were records for 530 (52.22%) small institutions, 87 (8.57%) medium institutions, 82 (8.08%) large institutions, and 19 (1.87%) very large institutions. In Southern Europe, there were 466 (72.47%) small institutions, 68 (10.58%) medium institutions, 53 (8.24%) large institutions, and 28 (4.35%) very large institutions, while in Middle Eastern Europe, we mapped 100 (48.31%) small institutions, 31 (14.98%) medium institutions, 35 (16.91%) large institutions, and 33 (15.94%) very large institutions.

**TABLE 2:** Institution legal status and institution category distribution in each geographical area.

		*Dimension*									
		Small		Medium		Big		Very big		ND	
		*N*	Row %	*N*	Row %	*N*	Row %	*N*	Row %	*N*	Row %
*Geographical area*	Northern Europe	368	66.55	103	18.63	52	9.40	2	0.36	28	5.06
	Eastern Europe	398	70.19	42	7.41	24	4.23	2	0.35	101	17.81
	Western Europe	530	52.22	87	8.57	82	8.08	19	1.87	297	29.26
	Southern Europe	466	72.47	68	10.58	53	8.24	28	4.35	28	4.35
	Middle Eastern Europe	100	48.31	31	14.98	35	16.91	33	15.94	8	3.86
***Tot***		*1,862*	*62.38*	*331*	*11.09*	*246*	*8.24*	*84*	*2.81*	*462*	*15.48*

		**Legal status**									
		**Public**		**Private**		**Private government- dependent**		**ND**			
		***N***	**%**	***N***	**%**	***N***	**%**	***N***	**%**		

*Geographical area*	Northern Europe	203	36.84	143	25.95	205	37.21	0	0.00		
	Eastern Europe	295	52.12	244	43.11	17	3.00	10	1.77		
	Western Europe	646	64.47	206	20.56	87	8.68	63	6.29		
	Southern Europe	339	55.57	248	40.66	22	3.61	1	0.16		
	Middle Eastern Europe	118	57.00	89	43.00	0	0.00	0	0.00		
***Tot***		*1,601*	*54.53*	*930*	*31.68*	*331*	*11.27*	*74*	*2.52*		

		**Institution category**									
		**University**		**University of applied sciences**		**Other**		**ND**			
		***N***	**%**	***N***	**%**	***N***	**%**	***N***	**%**		

*Geographical area*	Northern Europe	233	42.13	131	23.69	189	34.18	0	0.00		
	Eastern Europe	312	55.12	17	3.00	228	40.28	9	1.59		
	Western Europe	454	44.95	359	35.54	133	13.17	64	6.34		
	Southern Europe	418	68.52	96	15.74	94	15.41	2	0.33		
	Middle Eastern Europe	180	86.96	15	7.25	12	5.80	0	0.00		
***Tot***		*1,597*	*54.21*	*618*	*20.98*	*656*	*22.27*	*75*	*2.55*		

### Legal Statuses

As detailed in Table 2, in Northern Europe, we mapped 203 (36.84%) public institutions, 143 (25.95%) private institutions, and 205 (37.21%) private government-dependent institutions. In Eastern Europe, we found listings for 295 (52.12%) public institutions, 244 (43.11%) private institutions, and 17 (3.00%) private government-dependent institutions. Regarding Western Europe, there were records for 646 (64.47%) public institutions, 206 (20.56%) private institutions, and 87 (8.68%) private government-dependent institutions. In Southern Europe, there were 339 (55.57%) public institutions, 248 (40.66%) private institutions, and 22 (3.61%) private government-dependent institutions, while in Middle Eastern Europe, we mapped 118 (57.00%) public institutions and 89 (43.00%) private institutions.

### Institution Category

As also shown in Table 2, in Northern Europe, we mapped institution category for 233 (42.13%) universities, 131 (23.69%) universities of applied sciences, and 189 other types of institutions (34.18%). For Eastern Europe, we found 312 (55.12%) universities, 17 (3.00%) universities of applied sciences, and 228 (40.28%) other types of institutions. In respect of Western Europe, there were listed 454 (44.95%) universities, 359 (35.54%) universities of applied sciences, and 133 (13.17%) other types of institutions. In Southern Europe, we found records for 418 (68.52%) universities, 96 (15.74%) universities of applied sciences, and 94 (15.41%) other types of institutions. Lastly, in Middle Eastern Europe, there were 180 (86.96%) universities, 15 (7.25%) universities of applied sciences, and 12 (5.80%) other types of institutions.

### Availability of Counseling Centers and Services

We found available online data for 2,919 (97.79%) of the 2,985 institutions that we mapped (Table 3). As indicated in Table 4, the majority of European institutions reported having psychological centers or providing associated students psychological services, χ^2^ = 24.42, *p* < 0.001. Nonetheless, there were statistically significant differences regarding the presence of such provision between the European geographical areas, χ^2^ = 93.84, *p* < 0.001. In particular, in Northern Europe, Western Europe and Middle-Eastern Europe, most of the institutions reported having a student psychological center (respectively, χ^2^ = 69.77, *p* < 0.001, χ^2^ = 26.68, *p* < 0.001, and χ^2^ = 4.37, *p* = 0.037). However, most of the Eastern institutions did not report such services on their websites (χ^2^ = 15.08, *p* < 0.001). In Southern Europe, there were equal numbers of institutions that reported and did not report the provision of student psychological services.

**TABLE 3:** Online data available for every institutions in different European geographical areas.

		*Accessible information*		*Not accessible information*	
		*n*	Row %	*n*	Row %
*Geographical area*	Northern Europe	545	98.55	8	1.45
	Eastern Europe	549	96.83	18	3.17
	Western Europe	1,008	99.31	7	0.69
	Southern Europe	611	95.02	32	4.98
	Middle Eastern Europe	206	99.52	1	0.48
***Tot***		*2,919*	*97.79*	*66*	*2.21*

**TABLE 4:** Presence of student counseling services in different European geographical areas.

		*Students counseling services*				χ^2^	*df*	*p*
		*Not reported*		*Reported*				
		*n*	Row %	*n*	Row %			
*Geographical area*	Northern Europe	175	32.11	370	67.89	69.77	1	<0.001
	Eastern Europe	320	58.29	229	41.71	15.08	1	<0.001
	Western Europe	422	41.87	586	58.13	26.68	1	<0.001
	Southern Europe	321	52.54	290	47.46	1.57	1	0.210
	Middle Eastern Europe	88	42.72	118	57.28	4.37	1	0.037
***Tot***		*1,326*	*45.43*	*1,593*	*54.57*	*24.42*	*1*	<*0.001*

χ^2^ = 93.84, *df* = 1, *p* < 0.001.

### Presence of Students’ Psychological Centers According to Institution Dimension and Geographical Area

Table 5 summarizes the presence of student counseling services in institutions with different dimensions for the 2,462 institutions with complete information pertaining to both student centers and dimension. Throughout Europe, the percentage of institutions reporting student counseling centers differs according to the institutions’ various legal statuses (χ^2^ = 242.68, *p* < 0.001). In particular, the percentage of medium institutions (χ^2^ = 91.82, *p* < 0.001), large institutions (χ^2^ = 84.49, *p* < 0.001), and very large institutions (χ^2^ = 14.94, *p* < 0.001) reporting students’ psychological services is higher than the total percentage for Europe, while the percentage of small institutions reporting students psychological services is lower than the total percentage for Europe (χ^2^ = 48.48, *p* < 0.001).

**TABLE 5:** Presence of student counseling centers in institutions with different legal status in each European geographical area.

Geographical area			Students psychological centers				χ^2^	df	*p*
			*Not reported*		*Reported*				
			*n* [expected]	Row %	*n* [expected]	Row %			
*Northern Europe*	Dimension	Small	152 [110.81]	42.11	209 [250.19]	57.89	22.09	1	<0.001
		Medium	5 [31.62]	4.85	98 [71.38]	95.15	32.34	1	<0.001
		Large	1 [15.96]	1.92	51 [36.04]	98.08	20.23	1	<0.001
		Very large	1 [0.61]	50.00	1 [1.39]	50.00	0.36	1	0.549
	*Tot*		*159*	*30.69*	*359*	*69.31*			
χ^2^ = *75.01, df*= *3, p*< *0.001*									
*Eastern Europe*	*Dimension*	Small	255 [238.12]	66.93	126 [142.88]	33.07	3.19	1	0.074
		Medium	18 [25.63]	43.90	23 [15.37]	56.10	6.06	1	0.014
		Large	7 [15.00]	29.17	17 [9.00]	70.83	11.38	1	0.001
		Very large	0 [1.25]	0.00	2 [.75]	100.00	3.33	1	0.068
	*Tot*		*280*	*62.50*	*168*	*37.50*			
χ^2^ = *23.95, df*= *3, p*< *0.001*									
*Western Europe*	*Dimension*	Small	204 [160.47]	38.64	324 [367.53]	61.36	16.96	1	<0.001
		Medium	5 [26.44]	5.75	82 [60.56]	94.25	24.98	1	<0.001
		Large	8 [24.92]	9.75	74 [57.08]	90.24	16.50	1	<0.001
		Very large	0 [5.17]	0.00	17 [11.83]	100.00	7.43	1	0.006
	*Tot*		*217*	*30.39*	*497*	*69.61*			
χ^2^ = *65.87, df*= *3, p*< *0.001*									
*Southern Europe*	*Dimension*	Small	274 [224.49]	62.70	163 [212.51]	37.30	22.45	1	<0.001
		Medium	11 [34.42]	16.42	56 [32.58]	83.58	32.77	1	<0.001
		Large	11 [26.71]	21.15	41 [25.29]	78.85	19.00	1	<0.001
		Very large	4 [14.38]	14.29	24 [13.62]	85.71	15.40	1	0.001
	*Tot*		*300*	*51.37*	*284*	*48.63*			
χ^2^ = *89.64, df*= *3, p*< *0.001*									
*Middle Eastern Europe*	*Dimension*	Small	46 [41.92]	46.00	54 [58.08]	54.00	0.68	1	0.408
		Medium	14 [12.99]	45.16	17 [18.01]	54.84	0.14	1	0.713
		Large	11 [14.67]	31.43	24 [20.33]	68.57	1.58	1	0.209
		Very large	12 [13.41]	37.50	20 [18.59]	62.50	0.26	1	0.613
	*Tot*		*83*	*41.92*	*115*	*58.08*			
χ^2^ = *2.66, df*= *3, p*= *0.448*									
*All European areas*	*Dimension*	Small	931 [762.58]	51.52	876 [1,044.42]	48.48	64.36	1	<0.001
		Medium	53 [138.84]	16.11	276 [190.16]	83.89	91.82	1	<0.001
		Large	38 [103.39]	15.51	207 [141.61]	84.49	71.55	1	<0.001
		Very large	17 [34.18]	20.99	64 [46.82]	79.01	14.94	1	<0.001
	*Tot*		*1,039*	*42.20*	*1,423*	*57.80*			

χ^2^ = *242.68, df*= *3, p*< *0.001*

Regarding the different European geographical areas, in Northern Europe, the percentage of institutions reporting student counseling centers differs according to institutions’ dimensions (χ^2^ = 75.01, *p* < 0.001). For example, the percentage of medium (χ^2^ = 32.34, *p* < 0.001) and large (χ^2^ = 20.23, *p* < 0.001) institutions reporting students’ psychological centers is higher than the total percentage in that area, while the percentage of small institutions reporting such services is lower (χ^2^ = 22.09, *p* < 0.001). Likewise, in Western Europe, the percentage of institutions reporting student counseling centers differs according to legal status (χ^2^ = 65.87, *p* < 0.001). In particular, the percentage of medium χ^2^ = 24.98, *p* < 0.001, large (χ^2^ = 16.50, *p* < 0.001), and very large (χ^2^ = 7.43, *p* = 0.006) institutions reporting such services is higher than the total percentage for the area, while the percentage of small institutions reporting such services is lower (χ^2^ = 16.96, *p* < 0.001). In Southern Europe, also, the percentage of institutions reporting students’ psychological centers differs according to their dimensions (χ^2^ = 89.64, *p* < 0.001). Specifically, the percentage of medium (χ^2^ = 32.77, *p* < 0.001), large (χ^2^ = 19.00, *p* < 0.001) and very large (χ^2^ = 15.40, *p* < 0.001) institutions reporting such services is higher than the total percentage for that area, while the percentage of small institutions reporting such services is lower (χ^2^ = 22.45, *p* < 0.001). In Eastern Europe, also, the percentage of institutions reporting student counseling centers differs according to an institution’s dimension (χ^2^ = 23.95, *p* < 0.001). In particular, the percentage of medium (χ^2^ = 6.06, *p* = 0.014) and large (χ^2^ = 11.38, *p* = 0.001) institutions reporting students’ psychological centers is higher than the total percentage in that area. By contrast, though, in Middle Eastern Europe, the percentage of institutions reporting students’ psychological services was found not to differ according to an institution’s dimension (χ^2^ = 2.66, *p* = 0.448).

### Presence of Students’ Psychological Centers According to Legal Status and Geographical Area

Table 6 summarizes the presence of student counseling services in institutions with different legal statuses within each European geographical area for the 2,826 institutions with complete information pertaining to both student centers and dimensions. Throughout Europe, the percentage of institutions reporting student counseling centers differs according to the institutions’ various legal statuses (χ^2^ = 154.88, *p* < 0.001). In particular, the percentage of public (χ^2^ = 5.66, *p* = 0.017) and private government-dependent (χ^2^ = 77.94, *p* < 0.001) institutions reporting students psychological services is higher than the total percentage for Europe, while the percentage of private institutions reporting such services is lower (χ^2^ = 71.28, *p* < 0.001).

**TABLE 6:** Presence of student counseling services in different institution category in each European geographical area.

Geographical area			Students psychological centers				χ^2^	df	*p*
			*Not reported*		*reported*				
			*n* [expected]	Row %	*n* [expected]	Row %			
*Northern Europe*	*Legal status*	Public	71 [64.78]	35.32	130 [136.22]	64.68	0.88	1	0.348
		Private	91 [44.80]	65.47	48 [94.20]	34.53	70.30	1	<0.001
		Private government- dependent	13 [65.42]	6.40	190 [137.58]	93.60	61.98	1	<0.001
	*Tot*		*175*	*32.23*	*368*	*67.77*			
χ^2^ = *133.18, df*= *2, p*< *0.001*									
*Eastern Europe*	*Legal status*	Public	156 [168.18]	53.79	134 [121.82]	46.21	2.10	1	0.147
		Private	143 [134.54]	61.64	89 [97.46]	38.36	1.27	1	0.261
		Private government- dependent	13 [9.28]	81.25	3 [6.72]	18.75	3.55	1	0.060
	*Tot*		*312*	*57.99*	*226*	*42.01*			
χ^2^ = *6.92, df*= *2, p*= *0.031*									
*Western Europe*	*Legal status*	Public	218 [249.33]	33.85	426 [394.67]	66.15	6.42	1	0.011
		Private	112 [78.98]	54.90	92 [125.02]	45.10	22.53	1	<0.001
		Private government- dependent	32 [33.68]	36.78	55 [53.32]	63.22	0.14	1	0.712
	*Tot*		*362*	*38.72*	*573*	*61.28*			
χ^2^ = *29.09, df*= *2, p*< *0.001*									
*Southern Europe*	*Legal status*	Public	151 [175.26]	45.07	184 [159.74]	54.93	7.04	1	0.008
		Private	157 [129.23]	63.56	90 [117.77]	36.44	12.52	1	<0.001
		Private government- dependent	8 [11.51]	36.36	14 [10.49]	63.64	2.25	1	0.134
	*Tot*		*316*	*52.32*	*288*	*47.68*			
χ^2^ = *21.81, df*= *2, p*< *0.001*									
*Middle Eastern Europe*	*Legal status*	Public	61 [50.41]	51.69	57 [67.59]	48.31	3.88	1	0.049
		Private	27 [37.59]	30.68	61 [50.41]	69.32	5.21	1	0.023
	*Tot*		*88*	*42.72*	*118*	*57.28*			
χ^2^ = *9.860, df*= *1, p*= *0.002*									
*All European areas*	*Legal status*	Public	657 [704.09]	41.37	931 [883.91]	58.63	5.66	1	0.017
		Private	530 [403.48]	58.24	380 [506.52]	41.76	71.28	1	<0.001
		Private government- dependent	66 [145.43]	20.12	262 [182.57]	79.88	77.94	1	<0.001
	*Tot*		*1,253*	*44.34*	*1,573*	*55.66*			

χ^2^ = *154.88, df*= *2, p*< *0.001*

Regarding the different European geographical areas, in Northern Europe, the percentage of institutions reporting students’ psychological centers differs according to the institutions’ legal status (χ^2^ = 133.18, *p* < 0.001). For example, the percentage of private institutions reporting students’ psychological centers is lower than the total percentage in that geographical area (χ^2^ = 70.30, *p* < 0.001), while the percentage of private government-dependent institutions reporting such services is higher (χ^2^ = 61.98, *p* < 0.001). Likewise, in Western Europe, the percentage of institutions reporting students’ psychological centers differs according to legal status (χ^2^ = 29.09, *p* < 0.001). In particular, the percentage of public institutions reporting such provision is higher than the total percentage for the area overall (χ^2^ = 6.42, *p* = 0.011), while the percentage of private institutions is lower (χ^2^ = 22.53, *p* < 0.001). A similar pattern can be seen in Southern Europe where the percentage of institutions reporting students’ psychological centers also differs according to legal status (χ^2^ = 21.81, *p* < 0.001). Specifically, the percentage of private institutions reporting students’ psychological centers is lower than the total percentage for that area (χ^2^ = 12.52, *p* < 0.001), while the percentage of public institutions reporting such services is higher (χ^2^ = 7.04, *p* = 0.008). In Middle Eastern Europe, also, the percentage of institutions reporting students’ psychological centers differs according to the institutions’ legal status (χ^2^ = 9.10, *p* < 0.001). However, in this area, the percentage of private institutions reporting students’ psychological centers is higher than the total percentage of the area (χ^2^ = 5.21, *p* = 0.023), while the percentage of public institutions reporting them is lower than the total percentage (χ^2^ = 3.88, *p* = 0.049). In Eastern Europe, the percentage of institutions reporting students’ psychological centers differs according to legal status (χ^2^ = 6.92, *p* = 0.031); however, no statistically significant differences were found for what concerns each legal status.

### Presence of Students’ Psychological Centers According to Institution Category and Geographical Area

Table 7 the presence of students’ psychological services across different institution categories within each European geographical area for the 2,835 institutions featuring complete information pertaining to both student centers and institution category. We found that throughout Europe, the percentage of institutions reporting students’ psychological centers differs according to the institution category (χ^2^ = 130.17, *p* < 0.001). In particular, the percentage of universities and universities of applied sciences reporting students’ psychological centers was higher compared to the general European percentage (respectively, χ^2^ = 9.90, *p* = 0.002, and χ^2^ = 24.61, *p* < 0.001), while the percentage of other institutions reporting students’ psychological centers was lower (χ^2^ = 95.68, *p* < 0.001).

**TABLE 7:** Effect estimates of independent variables on the availability of student counseling centers.

Geographical area			Students psychological centers				χ^2^	df	*p*
			Not reported		Reported				
			*n* [expected]	Row %	*n* [expected]	Row %			
*Northern Europe*	*Institution category*	University	21 [74.50]	9.05	211 [157.50]	90.95	56.59	1	<0.001
		University of applied sciences	54 [40.46]	42.86	72 [85.54]	57.14	6.67	1	0.010
		Other	100 [60.05]	53.48	87 [126.95]	46.52	39.15	1	<0.001
	*Tot*		*175*	*32.11*	*370*	*67.89*			
χ^2^ = *102.42, df*= *2, p*< *0.001*									
*Eastern Europe*	*Institution category*	University	146 [177.70]	47.71	160 [128.30]	52.29	13.49	1	<0.001
		University of applied sciences	8 [8.71]	53.33	7 [6.29]	46.67	0.14	1	0.710
		Other	159 [126.59]	72.94	59 [91.41]	27.06	19.79	1	<0.001
	*Tot*		*313*	*58.07*	*226*	*41.93*			
χ^2^ = *33.41, df*= *2, p*< *0.001*									
*Western Europe*	*Institution category*	University	210 [174.75]	46.56	241 [276.25]	53.44	11.61	1	0.001
		University of applied sciences	83 [138.72]	23.18	275 [219.28]	76.82	36.54	1	<0.001
		Other	72 [51.53]	54.14	61 [81.47]	45.86	13.28	1	<0.001
	*Tot*		*365*	*38.75*	*577*	*61.25*			
χ^2^ = *61.41, df*= *2, p*< *0.001*									
*Southern Europe*	*Institution category*	University	191 [217.84]	45.80	226 [199.16]	54.20	6.92	1	0.009
		University of applied sciences	58 [49.63]	61.05	37 [45.37]	38.95	2.96	1	0.086
		Other	66 [47.54]	72.53	25 [43.46]	27.47	15.01	1	<0.001
	*Tot*		*315*	*52.24*	*288*	*47.76*			
χ^2^ = *24.89, df*= *2, p*< *0.001*									
*Middle Eastern Europe*	*Institution category*	University	72 [76.47]	40.22	107 [102.53]	59.78	0.46	1	0.499
		University of applied sciences	6 [6.41]	40.00	9 [8.59]	60.00	0.05	1	0.831
		Other	10 [5.13]	83.33	2 [6.87]	16.67	8.08	1	0.005
	*Tot*		*88*	*42.72*	*118*	*57.28*			
χ^2^ = *8.59, df*= *1, p*= *0.014*									
*All European areas*	*Institution category*	University	640 [702.21]	40.38	945 [882.79]	59.62	9.90	1	0.002
		University of applied sciences	209 [269.81]	34.32	400 [339.19]	65.68	24.61	1	<0.001
		Other	407 [283.98]	63.49	234 [357.02]	36.51	95.68	1	<0.001
	*Tot*		*1,256*	*44.30*	*1,579*	*55.70*			

χ^2^ = *130.17, df*= *2, p*< *0.001*

Focusing on the individual geographical areas, in Northern Europe, the percentage of institutions reporting students’ psychological centers can be seen to differ according to institution category (χ^2^ = 102.42, *p* < 0.001). In particular, the percentage of universities of applied sciences and other institutions reporting students’ psychological centers is lower compared to the total percentage in that area (respectively, χ^2^ = 6.67, *p* = 0.010, and χ^2^ = 39.15, *p* < 0.001), while the percentage of universities reporting such services is higher (χ^2^ = 56.59; *p* < 0.001). In Eastern Europe, also, the percentage of institutions reporting students’ psychological centers differs according to the institution category (χ^2^ = 33.41, *p* < 0.001). Specifically, the percentage of universities reporting students’ psychological centers is higher compared to the total percentage in that area (χ^2^ = 13.49, *p* < 0.001), while the percentage of other institutions reporting students’ psychological centers is lower (χ^2^ = 19.79, *p* < 0.001). Regarding Western Europe, the percentage of institutions reporting students’ psychological centers differs according to the institution category as well (χ^2^ = 61.41, *p* < 0.001). Here, the percentage of universities of applied sciences reporting students’ psychological centers is higher than the total percentage in Western Europe overall (χ^2^ = 36.54, *p* < 0.001), while the percentage of universities and other institutions reporting students’ psychological centers is lower than the total percentage (respectively, χ^2^ = 11.61, *p* = 0.001, and χ^2^ = 13.28, *p* < 0.001). In respect of Southern Europe, the percentage of institutions reporting students’ psychological centers also can be seen to differ according to institution category (χ^2^ = 24.89, *p* < 0.001). In this case, the percentage of universities reporting students’ psychological centers is higher compared to the total percentage found in the area (χ^2^ = 6.92, *p* = 0.009), while the percentage of other institutions reporting students’ psychological centers is lower than the total percentage (χ^2^ = 15.01, *p* < 0.001). Finally, in Middle Eastern Europe, the percentage of institutions reporting students’ counseling centers also differs according to an institutions’ category (χ^2^ = 8.59, *p* = 0.014). Of particular note in this instance is the observation that the percentage of other institutions reporting students’ psychological centers is lower than the total percentage for that area (χ^2^ = 8.08, *p* = 0.005).

### Predictors of the Presence of Students’ Psychological Centers

Table 8 shows the results of the logistic regression analysis. The logistic regression model was found to be statistically significant, χ^2^ = 485.56, *p* < 0.001. The model explained 24% (Nagelkerke’s R^2^) of the variance in the presence of students’ psychological centers and correctly classified 69.19% of cases. Regarding dimensions, medium institutions showed a higher probability of reporting students’ psychological centers than small institutions, the odds ratio (OR) = 4.22, 95% CI [3.03, 5.88], *p* < 0.001, as well as large institutions, OR = 4.50, 95% CI [3.07, 6.61], *p* < 0.001, and very large institutions, OR = 4.22, 95% CI [2.37, 7.53], *p* < 0.001. Concerning legal status, private government-dependent institutions showed a higher probability of reporting students’ psychological centers than public institutions, OR = 2.48, 95% CI [1.75, 3.51], *p* < 0.001. Regarding the category of institution, other institutions exhibited a lower likelihood of reporting students’ psychological centers than universities, OR = 0.47, 95% CI [0.36,0.60], *p* < 0.001. With respect to geographical areas, Eastern European institutions demonstrated a lower probability toward reporting students’ psychological centers than Northern European institutions, OR = 0.37, 95% CI [0.27,0.50], *p* < 0.001, as well as Southern European institutions, OR = 0.41, 95% CI [0.31,0.55], *p* < 0.001, and Middle Eastern European institutions, OR = 0.42, 95% CI [0.28,0.62], *p* < 0.001.

**TABLE 8:** Service offered by students counseling centers in different European areas.

	*B*	SE	Wald	df	OR	95% CI		*p*
						Lower	Lower	
Dimension: small			124.66	3.00	Ref			<0.001
Dimension: medium	1.44	0.17	72.37	1.00	4.22	3.03	5.88	<0.001
Dimension: big	1.50	0.20	59.14	1.00	4.50	3.07	6.61	<0.001
Dimension: very big	1.44	0.30	23.76	1.00	4.22	2.37	7.53	<0.001
Legal status: public			27.75	2.00				<0.001
Legal status: private	0.00	0.11	0.00	1.00	1.00	0.81	1.24	0.988
Legal status: private government-dependent	0.91	0.18	26.19	1.00	2.48	1.75	3.51	<0.001
Institution category: university			39.79	2.00	Ref			<0.001
Institution category: University of Applied Sciences	–0.10	0.13	0.64	1.00	0.90	0.70	1.16	0.424
Institution category: other	–0.76	0.13	36.57	1.00	0.47	0.36	0.60	<0.001
Geographical area: Northern Europe			94.91	4.00	Ref			<0.001
Geographical area: Eastern Europe	–0.99	0.15	41.24	1.00	0.37	0.27	0.50	<0.001
Geographical area: Western Europe	0.11	0.14	0.62	1.00	1.12	0.85	1.48	0.430
Geographical area: Southern Europe	–0.89	0.15	35.06	1.00	0.41	0.31	0.55	<0.001
Geographical area: Middle Eastern Europe	–0.88	0.21	17.93	1.00	0.42	0.28	0.62	<0.001
Costant	0.57	0.14	15.97	1.00	1.76			<0.001

Model χ^2^ = 485.56, p < 0.001. Nagelkerke R^2^ = 0.24. Accuracy (%) = 69.19%.

### Types of Interventions

As shown in Table 9, we found that European tertiary education institutions provide a variety of levels and types of counseling and/or support for their students. Many institutions reported offering otherwise unspecified psychological services (*n* = 285; 17.44%), counseling services (*n* = 906, 55.45%), career counseling services (*n* = 576, 32.25%), counseling for disabled students (*n* = 682; 41.74%) or counseling for learning specific disabilities (*n* = 254, 15.54%). Other types of services reported were, for example, psychotherapy (*n* = 41; 2.51%), psychiatric services (*n* = 20; 1.22%), group counseling (*n* = 11;0.67%), and psychoeducational counseling (*n* = 17; 1.04%).

**TABLE 9:** Service offered by students counseling centers in different European areas.

Geographical area		Psychological service				Counseling		Psychotherapy		Psychiatric services		Career counseling	
		*N*		*%*		*N*	*%*	*N*	*%*	*N*	*%*	*N*	*%*
Northern Europe	Not reported	348		93.80		78	21.02	361	97.57	365	98.38	262	70.62
	Reported	23		6.20		293	78.98	9	2.43	6	1.62	109	29.38
Eastern Europe	Not reported	197		85.28		121	52.38	228	98.70	229	99.13	104	45.02
	Reported	34		14.72		110	47.62	3	1.30	2	0.87	127	54.98
Western Europe	Not reported	462		45.52		345	26.01	595	96.59	607	98.54	379	61.52
	Reported	154		15.17		271	43.99	21	3.41	9	1.46	237	38.47
Southern Europe	Not reported	235		78.86		145	48.66	291	97.65	296	99.33	219	73.49
	Reported	63		21.14		153	51.34	7	2.35	2	0.67	79	26.51
Middle Eastern Europe	Not reported	107		90.68		39	33.05	117	99.15	117	99.15	94	45.41
	Reported	11		9.32		79	66.95	1	0.85	1	0.85	24	11.59
All European areas	Not reported	1,349		82.56		728	44.55	1,592	97.43	1,614	98.78	1,058	64.75
	Reported	285		17.44		906	55.45	41	2.51	20	1.22	576	35.25

**Geographical area**		**Group**		**Counseling for**		**Psychoeducational**		**Counseling for**		**Sport psychology**		**Spiritual**	
		**counseling**		**disabled students**		**counseling**		**foreign students**		**counseling**		**counseling**	
		*N*	*%*	*N*	*%*	*N*	*%*	*N*	*%*	*N*	*%*	*N*	*%*

Northern Europe	Not reported	366	69.66	133	35.85	371	100	370	99.73	371	100	371	100
	Reported	5	1.34	238	64.15	0	0.00	1	0.27	0	0.00	0	0.00
Eastern Europe	Not reported	229	99.13	179	77.49	230	99.57	228	98.70	230	99.57	230	99.57
	Reported	2	0.87	52	22.51	1	0.43	3	1.30	1	0.43	1	0.43
Western Europe	Not reported	615	99.84	399	64.77	616	100	613	99.51	615	99.86	615	99.84
	Reported	1	0.16	217	35.23	0	0.00	3	0.49	1	0.16	1	0.16
Southern Europe	Not reported	297	99.66	175	58.72	282	94.63	294	98.66	298	100	298	100
	Reported	1	0.34	123	41.28	16	5.37	4	1.34	0	0.00	0	0.00
Middle Eastern Europe	Not reported	116	98.31	66	55.93	118	100	118	100	118	100	118	100
	Reported	2	1.69	52	44.07	0	0.00	0	0.00	0	0.00	0	0.00
All European areas	Not reported	1,623	99.33	952	58.26	1,617	98.96	1,623	99.33	1,632	99.88	1,632	99.88
	Reported	11	0.67	682	41.74	17	1.04	11	0.67	2	0.12	2	0.12

**Geographical area**		**Social**		**Health**		**Sexual**		**Counseling for learning**		**Phone**		**Language**	
		**counseling**		**counseling**		**counseling**		**specific disabilities**		**counseling**		**counseling**	
		***N***	***%***	***N***	***%***	***N***	***%***	***N***	***%***	***N***	***%***	***N***	***%***

Northern Europe	Not reported	369	99.46	369	99.46	371	100	217	39.24	370	99.73	371	100
	Reported	2	0.54	2	0.54	0	0.00	154	27.85	1	0.27	0	0.00
Eastern Europe	Not reported	231	100	231	100	231	100	229	99.13	231	100	230	99.57
	Reported	0	0.00	0	0.00	0	0.00	2	0.87	0	0.00	1	0.43
Western Europe	Not reported	605	98.21	615	99.84	615	99.84	594	96.43	615	99.84	615	99.84
	Reported	11	1.08	1	0.16	1	0.16	22	3.57	1	0.16	1	0.16
Southern Europe	Not reported	298	100	298	100	297	99.66	225	75.50	298	100	298	100
	Reported	0	0.00	0	0.00	1	0.34	73	24.50	0	0.00	0	0.00
Middle Eastern Europe	Not reported	118	100	118	100	118	100	115	97.46	118	100	118	100
	Reported	0	0.00	0	0.00	0	0.00	3	2.54	0	0.00	0	0.00
All European areas	Not reported	1,621	99.20	1,631	99.82	1,632	99.88	1,380	84.46	1,632	99.88	1,632	99.88
	Reported	13	0.80	3	0.18	2	0.12	254	15.54	2	0.12	2	0.12

In terms of the different European geographical areas, psychological services were reported by 23 institutions in Northern Europe (6.20%), 34 in Eastern Europe (14.72%), 154 in Western Europe (15.17%), 63 in Southern Europe (21.14%), and 11 in Middle Eastern Europe (9.32%). Counseling services were found to be available in 293 institutions in Northern Europe (78.98%), 110 in Eastern Europe (47.62%), 271 in Western Europe (43.99%), 153 in Southern Europe (51.34%), and 79 in Middle Eastern Europe (33.95%). Career counseling was reported in 109 institutions in Northern Europe (29.38%), 127 in Eastern Europe (54.98%), 237 in Western Europe (38.47%), 79 in Southern Europe (26.51%), and 24 in Middle Eastern Europe (11.59%). Group counseling was only available in five institutions in Northern Europe (1.34), two in Eastern Europe (0.87%), one in Western Europe (0.16%), one in Southern Europe (0.34%), and two in Middle Eastern Europe (1.69%). Psychotherapy was provided by nine institutions in Northern Europe (2.43%), three in Eastern Europe (1.30%), 21 in Western Europe (3.41%), seven in Southern Europe (2.35%), and by just one university in Middle Eastern Europe (0.85%%). Psychiatric services were found to be available in six institutions in Northern Europe (1.62%), two in Eastern Europe (0.87%), nine in Western Europe (1.46%), two in Southern Europe (0.67%), and only one in Middle Eastern Europe (0.85%).

## Discussion

University students’ distress is an issue of increasing concern for public health because of its adverse effects on affected individuals’ personal development and academic performance (Storrie et al., 2010; Vivekananda et al., 2011; Hohenshil et al., 2013; Biasi et al., 2017). Several prior studies have highlighted an intensified demand for student counseling (Royal College of Psychiatrists, 2011; Watkins et al., 2012; Holm-Hadulla and Koutsoukou-Argyraki, 2015). According to an annual report of the Center for Collegiate Mental Health [CCMH] (2020), the number of students who have requested access to university counseling services for psychopathological symptoms and emotional disturbances (e.g., anxiety and depression) has increased by 1.0% since 2001. Similarly, a 2018 World Health Organization survey of students in eight countries found that roughly one out of three students screened positive for a mental health disorder (Auerbach et al., 2018).

We are aware that much of this data comes from an American context. The low proportion of European studies on this topic may depend on the differences in respect to the great difference in enrollment rates between European and non-European countries. This makes it even more imperative to conduct a study that includes every European area.

Yet, our data showed that only nearly half of European tertiary education institutions offered students’ psychological services. In a recent “Trends” report, The European University Association underlined how the proposed increase in individual academic counseling has not occurred and that “students still rate academic counseling as one of the weakest aspects of their programs” (Amundsen and Haakstad, 2017). Consistently, Black (2020) underlined that student support, psychological services, and student mental well-being are the most prevalent areas of discussion among student officers and institutions, but at the same time, the traditional approaches currently employed by institutions to tackle these areas are no longer suitable.

Our data also indicates that different dimensions, legal statuses and categories of institution report varying percentages concerning the presence of students’ psychological centers. In particular, compared to small institutions, all other institutions showed a higher likelihood of reporting students’ psychological centers. Moreover, compared to public institutions, private government-dependent institutions showed a higher probability of having such services. Moreover, compared to universities, other institutions were less likely to report providing such centers. The percentage of students’ psychological centers reported also differed according to geographical area. Compared to Northern Europe, Eastern Europe, Southern Europe, and Middle Eastern Europe, they all had a lower likelihood of reporting having students’ psychological centers. In Northern Europe, Western Europe and Middle Eastern Europe, most of the institutions reported having a student psychological center. However, most of the Eastern institutions did not report such services on their websites, and in Southern Europe, there were equal numbers of institutions that reported and did not report the provision of students’ psychological services. Overall, between the different European geographical areas, there were found to be differences concerning the presence of students’ psychological services in institutions according to differing dimensions, legal statuses and institutional categories compared to the general European data. Such results underline an urgent need to promote international data sharing with respect to students’ psychological centers, so that a broad range of professionals and departments (e.g., academic departments, administrative offices, and institutions) can cooperate both nationally and internationally in order to attain a wider integration of tertiary education services based on the sharing of experiences, expertise, training, and policies.

Regarding the specific services offered by each institution, most of them reported counseling services, career counseling services, or not otherwise specified psychological services. Previous research has demonstrated the efficacy of these interventions, while the sustained growth in the number of students entering higher education has challenged student counseling services to demonstrate the effectiveness of the support offered (Randall and Bewick, 2016). Connell et al. (2008) highlighted the effectiveness of university counseling services in the United Kingdom. Moreover, regarding the issues that such support can help to address, a Danish research has found that counseling is mostly offered to students with personal problems, such as low self-esteem, anxiety and depression, and study-related problems (e.g., exam anxiety and difficulties in writing a thesis), and also issues concerning social security laws (Østergård et al., 2019). In our study, only a small number of European institutions reported on their websites that they offer more specialized services, such as psychotherapies or psychiatric treatments.

It is important to underline that in the current work, only a few institutions in Europe reported offering psychotherapies despite the widely established effectiveness of these types of counseling support for tertiary education students. Notable among such research are the findings that, following psychotherapy, students show increased levels of well-being and reduced levels of distress, affirming the feasibility of psychotherapy in improving students’ mental health regardless of the particular psychotherapeutic approach utilized (Pistorello et al., 2012; Mukuria et al., 2013; Monti et al., 2014; Beiter et al., 2015; Kim et al., 2016; Rapinesi et al., 2018). Currently, though, mental health services and support in tertiary education institutions typically follow an approach that gives priority to first-level interventions, with only small subsets of students receiving assistance (Conley et al., 2015). Just 20 to 40% of students who experience mental health problems seek treatment while attending tertiary education, and this rate is even lower for students in public institutions (Ashwood et al., 2015; Conley et al., 2015). Thus, many students who need mental health services are not getting them and cannot receive them within their tertiary education institution (Czyz et al., 2013). Increasing the provision of psychotherapy services could engage a larger number of students during a critical phase of their life, thereby addressing a significant public health problem among late adolescents and emerging adults (Cleary et al., 2011).

Students with psychiatric disorders, both treated and untreated, have been shown to have lower performance levels and higher dropout rates than their peers (Eisenberg et al., 2009). Correspondingly, prior research has illustrated the effectiveness of psychological services in assisting students with psychiatric disabilities through higher education (Manthey et al., 2015; Schindler et al., 2015). However, it is a cause for concern that, as we found in our study, only a small number of tertiary education institutions in Europe provide specific psychiatric care. Given that the duration of an untreated mental illness affects prognosis and social functioning in relation to other mental diseases (e.g., depression, bipolar disorder, and obsessive compulsive disorder), the lack of psychiatric services provided for tertiary education students represents an issue in respect of which the consequences cannot be underestimated (Altamura et al., 2010, 2015; Ghio et al., 2014; Oguchi et al., 2014).

There should also be improved counseling in relation to disability and learning-specific difficulties. Indeed, learning-difficulty-related symptoms impact academic performance and college completion levels (Cortiella and Horowitz, 2014) as well as psychosocial functioning within post-secondary education (Kreider et al., 2015). Moreover, with respect to students who are disabled, prior research has demonstrated that providing appropriate support and services on campus can improve their mental health as well as their academic outcomes (Emerson et al., 2009; Stumbo et al., 2009). Such findings should be taken into account, especially when considering that, compared to other students, students with disabilities report greater levels of anxiety and academic-related distress as well as higher rates of suicide ideation, suicide attempts, and non-suicidal self-injury (Coduti et al., 2016).

### Limitations and Future Directions

This study has some critical limitations to be considered. First, the data available online was either not exhaustive, or else we could not find available data, especially on non-English websites. Moreover, there can be discrepancies in the use of some specific words (e.g., counseling) between the various countries surveyed.

Future research is needed to overcome these limitations and to deepen our understanding of the specific characteristics of the services provided throughout Europe. It is of importance to contact each tertiary education institution directly in order to assess the effective presence or absence of students’ psychological centers and the services provided as well as some additional, detailed information, such as how many practitioners are involved in the service’s staff, their qualifications, how many sessions are provided for each intervention, and the most common issues that they have to face.

### Clinical Implications

Despite these limitations, the present study is a first attempt to underline the importance of mapping the student counseling centers and services available in European tertiary education institutions and to promote an international data sharing of these important insights. This would enable a systematic and continuous monitoring of students’ mental health, allowing tertiary education institutions to identify their students’ mental health needs, as well as assess and improve the efficacy of their existing counseling programs. Moreover, such an approach would reinforce the collaboration between counseling staff and other bureaus (academic departments, administrative offices, and institutions), in order to attain a wider integration of university services (Güneri et al., 2003).

## Conclusion

It is crucial that more comprehensive services are provided to support students with mental health concerns, and innovative institutional, curricular, and service developments will be instrumental in cultivating healthy academic communities (Manthey et al., 2015). Health and education professionals alike must assign appropriate importance to students’ mental health and begin investing in tertiary education institutions as key sites for mental health promotion. Investing in professionally trained staff and in the possibility of long-term treatment options would go a long way in supporting students who might not otherwise have access to treatment.

## Data Availability Statement

The raw data supporting the conclusions of this article will be made available by the authors, without undue reservation.

## Author Contributions

IGF contributed to the study design, analysis and interpretation of data, and the drafting and critical revision of the manuscript. MS contributed to the analysis and interpretation of data and drafting the manuscript. GC contributed to data analysis and to drafting the manuscript. AG was involved in the interpretation of data and providing an important clinical and intellectual contribution. All authors contributed to the article and approved the submitted version.

## Conflict of Interest

The authors declare that the research was conducted in the absence of any commercial or financial relationships that could be construed as a potential conflict of interest.

## Publisher’s Note

All claims expressed in this article are solely those of the authors and do not necessarily represent those of their affiliated organizations, or those of the publisher, the editors and the reviewers. Any product that may be evaluated in this article, or claim that may be made by its manufacturer, is not guaranteed or endorsed by the publisher.
