# Correction: Student counseling centers in Europe: a retrospective analysis

**DOI:** 10.3389/fpsyg.2025.1645087

**Published:** 2025-09-10

**Authors:** Isabella Giulia Franzoi, Maria Domenica Sauta, Giuliano Carnevale, Antonella Granieri

**Affiliations:** Department of Psychology, University of Turin, Turin, Italy

**Keywords:** emerging adulthood, Europe, mental health, students, student counseling centers

The wrong table was erroneously inserted for Table 1. The correct table is included below:

**Table 1:** Descriptive statistics of the institutions mapped in each country for every geographical area.

**Geographical area**		***N***	***%***
Northern Europe	Denmark	33	5.97
	Estonia	25	4.52
	Finland	41	7.41
	Iceland	7	1.27
	Ireland	25	4.52
	Latvia	44	7.96
	Lithuania	43	7.78
	Norway	38	6.87
	Sweden	37	6.69
	UK	260	47.02
	*Total*	*553*	*100.00*
Eastern Europe	Bulgaria	52	9.17
	Czech Republic	65	11.46
	Hungary	53	9.35
	Poland	270	47.62
	Romania	95	16.75
	Slovakia	32	5.64
	*Total*	*567*	*100.00*
Western Europe	Austria	70	6.90
	Belgium	63	6.21
	France	389	38.33
	Germany	400	39.41
	Liechtenstein	1	0.10
	Luxembourg	2	0.20
	Netherlands	55	5.42
	Switzerland	35	3.45
	*Total*	*1,015*	*100.00*
Southern Europe	Albania	40	6.22
	Croatia	37	5.75
	Greece	47	7.31
	Italy	216	33.59
	Macedonia	16	2.49
	Malta	2	0.31
	Montenegro	10	1.56
	Portugal	95	14.77
	Serbia	46	7.15
	Slovenia	52	8.09
	Spain	82	12.75
	*Total*	*643*	*100.00*
Middle Eastern Europe	Cyprus	26	12.56
	Turkey	181	87.44
	*Total*	*207*	*100.00*
**Total**		**2,985**	**100**

The wrong table was erroneously inserted for Table 2. The correct table is included below:

Table 2Institution legal status and institution category distribution in each geographical area.

**Table:** 

		**Dimension**									
		**Small**		**Medium**		**Big**		**Very big**		**ND**	
		***N***	**Row %**	***N***	**Row %**	***N***	**Row %**	***N***	**Row %**	***N***	**Row %**
*Geographical area*	Northern Europe	368	66.55	103	18.63	52	9.40	2	0.36	28	5.06
	Eastern Europe	398	70.19	42	7.41	24	4.23	2	0.35	101	17.81
	Western Europe	530	52.22	87	8.57	82	8.08	19	1.87	297	29.26
	Southern Europe	466	72.47	68	10.58	53	8.24	28	4.35	28	4.35
	Middle Eastern Europe	100	48.31	31	14.98	35	16.91	33	15.94	8	3.86
***Tot***		*1,862*	*62.38*	*331*	*11.09*	*246*	*8.24*	*84*	*2.81*	*462*	*15.48*

**Table:** 

		**Legal status**							
		**Public**		**Private**		**Private government-dependent**		**ND**	
		***N***	**%**	***N***	**%**	***N***	**%**	***N***	**%**
*Geographical area*	Northern Europe	203	36.84	143	25.95	205	37.21	0	0.00
	Eastern Europe	295	52.12	244	43.11	17	3.00	10	1.77
	Western Europe	646	64.47	206	20.56	87	8.68	63	6.29
	Southern Europe	339	55.57	248	40.66	22	3.61	1	0.16
	Middle Eastern Europe	118	57.00	89	43.00	0	0.00	0	0.00
***Tot***		*1,601*	*54.53*	*930*	*31.68*	*331*	*11.27*	*74*	*2.52*
		**Institution category**							
		**University**		**University of applied sciences**		**Other**		**ND**	
		***N***	**%**	***N***	**%**	***N***	**%**	***N***	**%**
*Geographical area*	Northern Europe	233	42.13	131	23.69	189	34.18	0	0.00
	Eastern Europe	312	55.12	17	3.00	228	40.28	9	1.59
	Western Europe	454	44.95	359	35.54	133	13.17	64	6.34
	Southern Europe	418	68.52	96	15.74	94	15.41	2	0.33
	Middle Eastern Europe	180	86.96	15	7.25	12	5.80	0	0.00
***Tot***		*1,597*	*54.21*	*618*	*20.98*	*656*	*22.27*	*75*	*2.55*

The wrong table was erroneously inserted for Table 3. The correct table is included below:

**Table 3:** Online data available for every institutions in different European geographical areas.

		***Accessible information***		***Not accessible information***	
		***n***	**Row %**	***n***	**Row %**
*Geographical area*	Northern Europe	545	98.55	8	1.45
	Eastern Europe	549	96.83	18	3.17
	Western Europe	1,008	99.31	7	0.69
	Southern Europe	611	95.02	32	4.98
	Middle Eastern Europe	206	99.52	1	0.48
***Tot***		*2,919*	*97.79*	*66*	*2.21*

The wrong table was erroneously inserted for Table 4. The correct table is included below:

**Table 4:** Presence of student counseling services in different European geographical areas.

		**Students counseling services**				**χ^2^**	**df**	***p***
		***Not reported***		***Reported***				
		***n***	**Row %**	***n***	**Row %**			
*Geographical area*	Northern Europe	175	32.11	370	67.89	69.77	1	<0.001
	Eastern Europe	320	58.29	229	41.71	15.08	1	<0.001
	Western Europe	422	41.87	586	58.13	26.68	1	<0.001
	Southern Europe	321	52.54	290	47.46	1.57	1	0.210
	Middle Eastern Europe	88	42.72	118	57.28	4.37	1	0.037
***Tot***		*1,326*	*45.43*	*1,593*	*54.57*	*24.42*	*1*	<*0.001*

χ^2^ = 93.84, df = 1, p < 0.001.

The wrong table was erroneously inserted for Table 5. The correct table is included below:

**Table 5:** Presence of student counseling centers in institutions with different legal status in each European geographical area.

**Geographical area**			**Students psychological centers**				**χ^2^**	**df**	***p***
			***Not reported***		***Reported***				
			***n*** **[expected]**	**Row %**	***n*** **[expected]**	**Row %**			
*Northern Europe*	Dimension	Small	152 [110.81]	42.11	209 [250.19]	57.89	22.09	1	<0.001
		Medium	5 [31.62]	4.85	98 [71.38]	95.15	32.34	1	<0.001
		Large	1 [15.96]	1.92	51 [36.04]	98.08	20.23	1	<0.001
		Very large	1 [0.61]	50.00	1 [1.39]	50.00	0.36	1	0.549
	*Tot*		*159*	*30.69*	*359*	*69.31*			
*χ^2^ = 75.01, df = 3, p < 0.001*									
*Eastern Europe*	*Dimension*	Small	255 [238.12]	66.93	126 [142.88]	33.07	3.19	1	0.074
		Medium	18 [25.63]	43.90	23 [15.37]	56.10	6.06	1	0.014
		Large	7 [15.00]	29.17	17 [9.00]	70.83	11.38	1	0.001
		Very large	0 [1.25]	0.00	2 [.75]	100.00	3.33	1	0.068
	*Tot*		*280*	*62.50*	*168*	*37.50*			
*χ^2^ = 23.95, df = 3, p < 0.001*									
*Western Europe*	*Dimension*	Small	204 [160.47]	38.64	324 [367.53]	61.36	16.96	1	<0.001
		Medium	5 [26.44]	5.75	82 [60.56]	94.25	24.98	1	<0.001
		Large	8 [24.92]	9.75	74 [57.08]	90.24	16.50	1	<0.001
		Very large	0 [5.17]	0.00	17 [11.83]	100.00	7.43	1	0.006
	*Tot*		*217*	*30.39*	*497*	*69.61*			
*χ^2^ = 65.87, df = 3, p < 0.001*									
*Southern Europe*	*Dimension*	Small	274 [224.49]	62.70	163 [212.51]	37.30	22.45	1	<0.001
		Medium	11 [34.42]	16.42	56 [32.58]	83.58	32.77	1	<0.001
		Large	11 [26.71]	21.15	41 [25.29]	78.85	19.00	1	<0.001
		Very large	4 [14.38]	14.29	24 [13.62]	85.71	15.40	1	0.001
	*Tot*		*300*	*51.37*	*284*	*48.63*			
*χ^2^ = 89.64, df = 3, p < 0.001*									
*Middle Eastern Europe*	*Dimension*	Small	46 [41.92]	46.00	54 [58.08]	54.00	0.68	1	0.408
		Medium	14 [12.99]	45.16	17 [18.01]	54.84	0.14	1	0.713
		Large	11 [14.67]	31.43	24 [20.33]	68.57	1.58	1	0.209
		Very large	12 [13.41]	37.50	20 [18.59]	62.50	0.26	1	0.613
	*Tot*		*83*	*41.92*	*115*	*58.08*			
*χ^2^ = 2.66, df = 3, p = 0.448*									
*All European areas*	*Dimension*	Small	931 [762.58]	51.52	876 [1,044.42]	48.48	64.36	1	<0.001
		Medium	53 [138.84]	16.11	276 [190.16]	83.89	91.82	1	<0.001
		Large	38 [103.39]	15.51	207 [141.61]	84.49	71.55	1	<0.001
		Very large	17 [34.18]	20.99	64 [46.82]	79.01	14.94	1	<0.001
	*Tot*		*1,039*	*42.20*	*1,423*	*57.80*			
*χ^2^ = 242.68, df = 3, p < 0.001*									

The wrong table was inserted for Table 6. The correct table is included below:

**Table 6:** Presence of student counseling services in different institution category in each European geographical area.

**Geographical area**			**Students psychological centers**				**χ^2^**	**df**	***p***
			***Not reported***		***reported***				
			***n*** **[expected]**	**Row %**	***n*** **[expected]**	**Row %**			
*Northern Europe*	*Legal status*	Public	71 [64.78]	35.32	130 [136.22]	64.68	0.88	1	0.348
		Private	91 [44.80]	65.47	48 [94.20]	34.53	70.30	1	<0.001
		Private government-dependent	13 [65.42]	6.40	190 [137.58]	93.60	61.98	1	<0.001
	*Tot*		*175*	*32.23*	*368*	*67.77*			
*χ^2^ = 133.18, df = 2, p < 0.001*									
*Eastern Europe*	*Legal status*	Public	156 [168.18]	53.79	134 [121.82]	46.21	2.10	1	0.147
		Private	143 [134.54]	61.64	89 [97.46]	38.36	1.27	1	0.261
		Private government-dependent	13 [9.28]	81.25	3 [6.72]	18.75	3.55	1	0.060
	*Tot*		*312*	*57.99*	*226*	*42.01*			
*χ^2^ = 6.92, df = 2, p = 0.031*									
*Western Europe*	*Legal status*	Public	218 [249.33]	33.85	426 [394.67]	66.15	6.42	1	0.011
		Private	112 [78.98]	54.90	92 [125.02]	45.10	22.53	1	<0.001
		Private government-dependent	32 [33.68]	36.78	55 [53.32]	63.22	0.14	1	0.712
	*Tot*		*362*	*38.72*	*573*	*61.28*			
*χ^2^ = 29.09, df = 2, p < 0.001*									
*Southern Europe*	*Legal status*	Public	151 [175.26]	45.07	184 [159.74]	54.93	7.04	1	0.008
		Private	157 [129.23]	63.56	90 [117.77]	36.44	12.52	1	<0.001
		Private government-dependent	8 [11.51]	36.36	14 [10.49]	63.64	2.25	1	0.134
	*Tot*		*316*	*52.32*	*288*	*47.68*			
*χ^2^ = 21.81, df = 2, p < 0.001*									
*Middle Eastern Europe*	*Legal status*	Public	61 [50.41]	51.69	57 [67.59]	48.31	3.88	1	0.049
		Private	27 [37.59]	30.68	61 [50.41]	69.32	5.21	1	0.023
	*Tot*		*88*	*42.72*	*118*	*57.28*			
	*χ^2^ = 9.860, df = 1, p = 0.002*									
*All European areas*	*Legal status*	Public	657 [704.09]	41.37	931 [883.91]	58.63	5.66	1	0.017
		Private	530 [403.48]	58.24	380 [506.52]	41.76	71.28	1	<0.001
		Private government-dependent	66 [145.43]	20.12	262 [182.57]	79.88	77.94	1	<0.001
	*Tot*		*1,253*	*44.34*	*1,573*	*55.66*			
*χ^2^ = 154.88, df = 2, p < 0.001*									

The wrong table was inserted for Table 7. The correct table is included below:

**Table 7:** Effect estimates of independent variables on the availability of student counseling centers.

**Geographical area**			**Students psychological centers**				**χ^2^**	**df**	***p***
			**Not reported**		**Reported**				
			***n*** **[expected]**	**Row %**	***n*** **[expected]**	**Row %**			
*Northern Europe*	*Institution category*	University	21 [74.50]	9.05	211 [157.50]	90.95	56.59	1	<0.001
		University of applied sciences	54 [40.46]	42.86	72 [85.54]	57.14	6.67	1	0.010
		Other	100 [60.05]	53.48	87 [126.95]	46.52	39.15	1	<0.001
	*Tot*		*175*	*32.11*	*370*	*67.89*			
*χ^2^ = 102.42, df = 2, p < 0.001*									
*Eastern Europe*	*Institution category*	University	146 [177.70]	47.71	160 [128.30]	52.29	13.49	1	<0.001
		University of applied sciences	8 [8.71]	53.33	7 [6.29]	46.67	0.14	1	0.710
		Other	159 [126.59]	72.94	59 [91.41]	27.06	19.79	1	<0.001
	*Tot*		*313*	*58.07*	*226*	*41.93*			
*χ^2^ = 33.41, df = 2, p < 0.001*									
*Western Europe*	*Institution category*	University	210 [174.75]	46.56	241 [276.25]	53.44	11.61	1	0.001
		University of applied sciences	83 [138.72]	23.18	275 [219.28]	76.82	36.54	1	<0.001
		Other	72 [51.53]	54.14	61 [81.47]	45.86	13.28	1	<0.001
	*Tot*		*365*	*38.75*	*577*	*61.25*			
*χ^2^ = 61.41, df = 2, p < 0.001*									
*Southern Europe*	*Institution category*	University	191 [217.84]	45.80	226 [199.16]	54.20	6.92	1	0.009
		University of applied sciences	58 [49.63]	61.05	37 [45.37]	38.95	2.96	1	0.086
		Other	66 [47.54]	72.53	25 [43.46]	27.47	15.01	1	<0.001
	*Tot*		*315*	*52.24*	*288*	*47.76*			
*χ^2^ = 24.89, df = 2, p < 0.001*									
*Middle Eastern Europe*	*Institution category*	University	72 [76.47]	40.22	107 [102.53]	59.78	0.46	1	0.499
		University of applied sciences	6 [6.41]	40.00	9 [8.59]	60.00	0.05	1	0.831
		Other	10 [5.13]	83.33	2 [6.87]	16.67	8.08	1	0.005
	*Tot*		*88*	*42.72*	*118*	*57.28*			
*χ^2^ = 8.59, df = 1, p = 0.014*									
*All European areas*	*Institution category*	University	640 [702.21]	40.38	945 [882.79]	59.62	9.90	1	0.002
		University of applied sciences	209 [269.81]	34.32	400 [339.19]	65.68	24.61	1	<0.001
		Other	407 [283.98]	63.49	234 [357.02]	36.51	95.68	1	<0.001
	*Tot*		*1,256*	*44.30*	*1,579*	*55.70*			
*χ^2^ = 130.17, df = 2, p < 0.001*									

The wrong table was inserted for Table 8. The correct table is included below:

**Table 8:** Service offered by students counseling centers in different European areas.

	***B***	**SE**	**Wald**	**df**	**OR**	**95% CI**		***p***
						**Lower**	**Lower**	
Dimension: small			124.66	3.00	Ref			<0.001
Dimension: medium	1.44	0.17	72.37	1.00	4.22	3.03	5.88	<0.001
Dimension: big	1.50	0.20	59.14	1.00	4.50	3.07	6.61	<0.001
Dimension: very big	1.44	0.30	23.76	1.00	4.22	2.37	7.53	<0.001
Legal status: public			27.75	2.00				<0.001
Legal status: private	0.00	0.11	0.00	1.00	1.00	0.81	1.24	0.988
Legal status: private government-dependent	0.91	0.18	26.19	1.00	2.48	1.75	3.51	<0.001
Institution category: university			39.79	2.00	Ref			<0.001
Institution category: University of Applied Sciences	−0.10	0.13	0.64	1.00	0.90	0.70	1.16	0.424
Institution category: other	−0.76	0.13	36.57	1.00	0.47	0.36	0.60	<0.001
Geographical area: Northern Europe			94.91	4.00	Ref			<0.001
Geographical area: Eastern Europe	−0.99	0.15	41.24	1.00	0.37	0.27	0.50	<0.001
Geographical area: Western Europe	0.11	0.14	0.62	1.00	1.12	0.85	1.48	0.430
Geographical area: Southern Europe	−0.89	0.15	35.06	1.00	0.41	0.31	0.55	<0.001
Geographical area: Middle Eastern Europe	−0.88	0.21	17.93	1.00	0.42	0.28	0.62	<0.001
Costant	0.57	0.14	15.97	1.00	1.76			<0.001

Model χ^2^ = 485.56, p < 0.001.

Nagelkerke R^2^ = 0.24.

Accuracy (%) = 69.19%.

The wrong table was inserted for Table 9. The correct table is included below:

Table 9Service offered by students counseling centers in different European areas.

**Table:** 

**Geographical area**		**Psychological service**		**Counseling**		**Psychotherapy**		**Psychiatric services**		**Career counseling**	
		***N***	***%***	***N***	***%***	***N***	***%***	***N***	***%***	***N***	***%***
Northern Europe	Not reported	348	93.80	78	21.02	361	97.57	365	98.38	262	70.62
	Reported	23	6.20	293	78.98	9	2.43	6	1.62	109	29.38
Eastern Europe	Not reported	197	85.28	121	52.38	228	98.70	229	99.13	104	45.02
	Reported	34	14.72	110	47.62	3	1.30	2	0.87	127	54.98
Western Europe	Not reported	462	45.52	345	26.01	595	96.59	607	98.54	379	61.52
	Reported	154	15.17	271	43.99	21	3.41	9	1.46	237	38.47
Southern Europe	Not reported	235	78.86	145	48.66	291	97.65	296	99.33	219	73.49
	Reported	63	21.14	153	51.34	7	2.35	2	0.67	79	26.51
Middle Eastern Europe	Not reported	107	90.68	39	33.05	117	99.15	117	99.15	94	45.41
	Reported	11	9.32	79	66.95	1	0.85	1	0.85	24	11.59
All European areas	Not reported	1,349	82.56	728	44.55	1,592	97.43	1,614	98.78	1,058	64.75
	Reported	285	17.44	906	55.45	41	2.51	20	1.22	576	35.25

**Table:** 

**Geographical area**		**Group counseling**		**Counseling for disabled students**		**Psychoeducational counseling**		**Counseling for foreign students**		**Sport psychology counseling**		**Spiritual counseling**	
		***N***	***%***	***N***	***%***	***N***	***%***	***N***	***%***	***N***	***%***	***N***	***%***
Northern Europe	Not reported	366	69.66	133	35.85	371	100	370	99.73	371	100	371	100
	Reported	5	1.34	238	64.15	0	0.00	1	0.27	0	0.00	0	0.00
Eastern Europe	Not reported	229	99.13	179	77.49	230	99.57	228	98.70	230	99.57	230	99.57
	Reported	2	0.87	52	22.51	1	0.43	3	1.30	1	0.43	1	0.43
Western Europe	Not reported	615	99.84	399	64.77	616	100	613	99.51	615	99.86	615	99.84
	Reported	1	0.16	217	35.23	0	0.00	3	0.49	1	0.16	1	0.16
Southern Europe	Not reported	297	99.66	175	58.72	282	94.63	294	98.66	298	100	298	100
	Reported	1	0.34	123	41.28	16	5.37	4	1.34	0	0.00	0	0.00
Middle Eastern Europe	Not reported	116	98.31	66	55.93	118	100	118	100	118	100	118	100
	Reported	2	1.69	52	44.07	0	0.00	0	0.00	0	0.00	0	0.00
All European areas	Not reported	1,623	99.33	952	58.26	1,617	98.96	1,623	99.33	1,632	99.88	1,632	99.88
	Reported	11	0.67	682	41.74	17	1.04	11	0.67	2	0.12	2	0.12
**Geographical area**		**Social counseling**		**Health counseling**		**Sexual counseling**		**Counseling for learning specific disabilities**		**Phone counseling**		**Language counseling**	
		***N***	***%***	***N***	***%***	***N***	***%***	***N***	***%***	***N***	***%***	***N***	***%***
Northern Europe	Not reported	369	99.46	369	99.46	371	100	217	39.24	370	99.73	371	100
	Reported	2	0.54	2	0.54	0	0.00	154	27.85	1	0.27	0	0.00
Eastern Europe	Not reported	231	100	231	100	231	100	229	99.13	231	100	230	99.57
	Reported	0	0.00	0	0.00	0	0.00	2	0.87	0	0.00	1	0.43
Western Europe	Not reported	605	98.21	615	99.84	615	99.84	594	96.43	615	99.84	615	99.84
	Reported	11	1.08	1	0.16	1	0.16	22	3.57	1	0.16	1	0.16
Southern Europe	Not reported	298	100	298	100	297	99.66	225	75.50	298	100	298	100
	Reported	0	0.00	0	0.00	1	0.34	73	24.50	0	0.00	0	0.00
Middle Eastern Europe	Not reported	118	100	118	100	118	100	115	97.46	118	100	118	100
	Reported	0	0.00	0	0.00	0	0.00	3	2.54	0	0.00	0	0.00
All European areas	Not reported	1,621	99.20	1,631	99.82	1,632	99.88	1,380	84.46	1,632	99.88	1,632	99.88
	Reported	13	0.80	3	0.18	2	0.12	254	15.54	2	0.12	2	0.12

The original article has been updated.

